# Prevalence and factors associated with psychological distress among key populations in Togo, 2017

**DOI:** 10.1371/journal.pone.0231726

**Published:** 2020-04-16

**Authors:** Martin Kouame Tchankoni, Fifonsi Adjidossi Gbeasor-Komlanvi, Alexandra Marie Bitty-Anderson, Essèboè Koffitsè Sewu, Wendpouiré Ida Carine Zida-Compaore, Ahmadou Alioum, Mounerou Salou, Claver Anoumou Dagnra, Didier Koumavi Ekouevi

**Affiliations:** 1 African Center for Epidemiology and Public Health Research (CARESP), Lomé, Togo; 2 Department of Public Health, University of Lomé, Faculty of Health Sciences, Lomé, Togo; 3 PACCI Program—ANRS Ivory Coast Site, Treichville University Hospital, Abidjan, Ivory Coast; 4 University of Bordeaux, Inserm Center, Bordeaux, France; 5 University of Bordeaux, Institute of Public Health Epidemiology Development (ISPED), Bordeaux, France; 6 Department of Basic Sciences, University of Lomé, Center for Molecular Biology and Immunology, Lomé, Togo; University of the Witwatersrand, SOUTH AFRICA

## Abstract

**Objectives:**

Mental health is a largely neglected issue among in Sub-Saharan Africa, especially among key populations at risk for HIV. The aim of this study was to estimate the prevalence of psychological distress (PD) and to assess the factors associated among males who have sex with males (MSM), female sex workers (FSW) and drug users (DU) in Togo in 2017.

**Study design:**

A cross-sectional bio-behavioral study was conducted in August and September 2017 using a respondent-driven sampling (RDS) method, in eight cities in Togo.

**Methods:**

A standardized questionnaire was used to record sociodemographic characteristics and sexual behaviors. The Alcohol Use Disorders Identification Test (AUDIT) and a subset of questions from the Tobacco Questions for Survey were used to assess alcohol and tobacco consumption respectively. PD was assessed with the Kessler Psychological Distress Scale. A blood sample was taken to test for HIV. Descriptive statistics, univariable and multivariable ordinal regression models were used for analysis.

**Results:**

A total of 2044 key populations including 449 DU, 952 FSW and 643 MSM with a median age of 25 years, interquartile range (IQR) [21–32] were recruited. The overall prevalence of mild PD among the three populations was 19.9% (95%CI = [18.3–21.8]) and was 19.2% (95%CI = [17.5–20.9]) for severe/moderate PD. HIV prevalence was 13.7% (95%CI = [12.2–15.2]). High age (≥ 25 years) [aOR = 1.24 (95% CI: 1.02–1.50)], being HIV positive [aOR = 1.80 (95% CI: 1.31–2.48)] and hazardous alcohol consumption [aOR = 1.52 (95% CI: 1.22–1.87)] were risk factors for PD. Secondary [aOR = 0.52 (95% CI: 0.42–0.64)] or higher [aOR = 0.46 (95% CI: 0.32–0.64)] education levels were protective factors associated with PD. FSW [OR = 0.55 (95% CI: 0.43–0.68)] and MSM [OR = 0.33 (95% CI: 0.24–0.44)] were less likely to report PD compared with DU.

**Conclusion and recommendations:**

This is the first study conducted among a large, nationally representative sample of key populations in Togo. The prevalence of PD is high among these populations in Togo and was associated to HIV infection. The present study indicates that mental health care must be integrated within health programs in Togo with a special focus to key populations through interventions such as social support groups.

## Introduction

Mental health disorders represent a growing public health challenge worldwide. According to the World Health Organization (WHO), mental health is a state of well-being in which every individual realizes his or her own potential, can cope with the normal stresses of life, can work productively and fruitfully, and is able to make a contribution to her or his community [1]. Between 1990 and 2010, the burden of mental health disorders, such as depression and other common mental disorders, alcohol-use/substance-use disorders, and psychoses, increased by 37.6% [2,3]. Overall, mental health disorders are key contributors to nearly 10 to 14% of the global burden of disease, including death and disability [3,4]. Several factors have been associated with mental health, including gender, marital status, education, tobacco use, partner control/abuse [5,6].

Some population subgroups, such as those at higher risk of HIV are particularly burdened with mental health issues. According to the UNAIDS, gay men and other men who have sex with men (MSM), female sex workers (FSW) and their clients, transgender people, people who inject drugs and prisoners and other incarcerated people are the main key population groups [7]. In a study conducted in the Netherlands in 1999, mental health disorders were more prevalent among homosexually active people compared with their heterosexually active counterparts [8]. In addition, Luo et al. in a meta-analysis, reported that the pooled lifetime prevalence of suicidal ideation among MSM was 34.9% [9]. Similarly, using the Center for Epidemiological Studies Short Depression Scale, FSW in the Dominican Republic were more frequently screened positive for depression compared with HIV negative women who were not FSW (70.2% vs 52.2%) [10]. Moreover, poor mental health has been identified as a key contributor to the HIV epidemic among key populations. Indeed, MSM who are mentally depressed engage in unprotected sex and substance abuse [11–13]. Several studies have demonstrated a significant association between depressive symptoms and HIV and other sexually transmitted infections (STI) among MSM [14–16]. Poor mental health is a major concern in sub-Saharan Africa which bears the heaviest burden of HIV/AIDS [17]. As an integral part of overall well-being, mental health is not on the front line in terms of priority for health practitioners and it has been a largely neglected issue among marginalized populations in the developing world [17,18].

The high rates of HIV/AIDS among MSM and other key populations have pushed preventive interventions to focus on the risks associated with HIV/AIDS transmission and this situation has led to mental health neglect [19]. In Togo, HIV prevalence among key populations ranges from 11% to 13% compared to 2.1% in the general population [20,21]. Although issues related to key populations have been extensively studied in Togo, to our knowledge, no data are available on mental health problems among these populations. The aim of this study was to assess the prevalence of and factors associated with PD among MSM, FSW and DU in Togo in 2017.

## Methods

### Study design and sampling

This study was a bio-behavioral cross-sectional study conducted from August to September 2017 in eight cities in Togo. Prior to the study, locations (associations and hot spots) specific to each group of key population were identified during preliminary visits with the help of leaders from these communities. DU and FSW were recruited in drug-dealing/consumption locations and brothels (licensed or not), respectively. MSM were recruited using a Respondent Driven Sampling (RDS) method. MSM community leaders were the first “seeds”. A total of 28 seeds were identified at first based on their roles in their community and on their representativeness. Each seed from the first wave selected had to represent at least one sub-group: the actives (the ones who penetrate during sex), the passives (the penetrated), bisexuals, gays, etc. Each participant was then given three coupons with a unique identification code to recruit three other seeds in their network until the required sample size for each group was reached. Inclusion criteria for the three groups were being 18 years or older, living/working/studying in Togo for a minimum of 3 months at the time of the study, and being in possession of a recruitment coupon. In addition to these criteria, criteria specific to MSM were having had anal sex with a man in the previous 12 months, for FSW having had sex in exchange for money as a compensation in the previous 12 months and for DU, consuming heroin, cocaine or hashish at the time of the study.

### Ethics approval and consent to participate

This study was approved by the “Comité de Bioéthique pour la Recherche en Santé” (Bioethics Committee for Health Research) from the Togo Ministry of Health (CBRS No. 18/2017/CBRS of June 22^nd^ 2017). Potential participants were informed about the study purpose and procedures, potential risks and protections, and compensation. Informed consent was documented with signed consent forms prior to participation.

### Data collection

After eligibility screening and written informed consent, a structured and standardized questionnaire was administered by trained study staff during a face-to-face interview. The Kessler PD Scale (K10) [22] was used to measure PD. The K10 is a 10-item screening tool which has been used in several countries and it has been validated in a low-income, African setting with strong validity and reliability [23,24].

Alcohol and tobacco consumption were also assessed using validated tools: the Alcohol Use Disorders Identification Test (AUDIT) [25] and a subset of the Tobacco Questions for Surveys [26] respectively. To collect information on socio-demographic characteristics, risky sexual behaviors, STIs, HIV prevention methods, HIV testing history, access to health care services, and HIV knowledge, items from the Family Health International (FHI) 360 validated guide for bio-behavioral surveys [27] were included in the questionnaire. The same questionnaires were used across the three populations with slight adaptations depending on the population.

### Laboratory testing

Two HIV rapid tests were performed using SD Biolane Duo VIH/Syphilis rapid test kits. For each participant, a 4 mL blood sample was collected and a third test, ImmunoComb II VIH1-2 Comfirm (Orgenics Ltd, Israël) was used for confirmation. All biological test analyses were completed in the principal HIV laboratory research unit, the Molecular Biology Laboratory (CBMI) at the University of Lomé.

### Scores and operational definitions

#### Psychological distress

The K10 has been examined and validated among several populations and aims at measuring anxiety and depression with a 10-item questionnaire, each question pertaining to an emotional state and a five-level response scale for each response. The score obtained from the scale allows categorization of participants into four ordinal categories of PD: severe (score ≥ 30), moderate (score: 25–29), mild (score: 20–24) and none (score < 20) [28]. In our study, due to the small number of participants falling in the severe category, we collapsed the Kessler PD scores into three categories as: severe/moderate (score ≥25), mild (score: 20–24) and none (score <20).

#### Alcohol consumption

To assess alcohol consumption, the AUDIT-C was used. The AUDIT-C is a screening tool used to identify persons who are hazardous drinkers or have active alcohol use disorders (including alcohol abuse or dependence). The AUDIT-C is a modified version of the 10 question AUDIT instrument and it proved to be as effective as the AUDIT [29]. As with the 10-item AUDIT, higher scores indicate more problematic alcohol use. The AUDIT-C is scored on a scale of 0–12. Each question has 5 answer choices and can obtain a score from 0 to 4. A score ≥5 for men and ≥4 for women indicates hazardous drinking, while a score of 0 indicates a non-drinker; moderate alcohol use lies in-between [25,30,31].

### Statistical analysis

We used a Partial Proportional Odds (PPO) model to assess factors associated to PD. Descriptive statistics were performed and results were presented with frequency tabulations and percentages. Prevalence rates were estimated with their 95% confidence interval. For model building, characteristics that had a p-value <0.20 in univariable analysis were considered for the full multivariable models, which were subsequently finalized using a stepwise, backward elimination approach (p-value <0.05). Predictor variables were selected as those found to be relevant according to the literature review. All computations were conducted using SAS version 9.4 (SAS Institute Inc., Cary, NC, USA).

## Results

### Sociodemographic and clinical characteristics

A total of 2044 key populations including 449 DU, 952 FSW and 643 MSM with a median age of 25 years, interquartile range (IQR) [21–32 years] were included. More than two-thirds (68.5%) of participants had at least a secondary education level and 15.7% were married or living with a partner. The HIV prevalence was 13.7% (95%CI = [12.2–15.2]) across the three key populations with the highest prevalence among MSM (21.6%; 95%CI = [18.5–25.0]). Other sociodemographic and clinical characteristics are summarized in Table 1.

**Table 1:** Sociodemographic and clinical characteristics of key populations, Togo 2017.

	DU (n = 449)	FSW (n = 952)	MSM (n = 643)	Total (= 2044)
**Age (years), median [IQR]**	29 [23–37]	26 [22–32]	23 [20–26]	25 [21–32]
**Marital status, n (%)**				
Married/Living with a partner	148 (33.0)	133 (14.0)	40 (6.2)	321 (15.7)
Not married	301 (67.0)	819 (86.0)	603 (93.8)	1723 (84.3)
**Education level, n (%)**				
Primary school	160 (35.6)	429 (45.1)	54 (8.4)	643 (31.5)
Secondary school	268 (59.7)	476 (50.0)	358 (55.7)	1102 (53.9)
College/University	21 (4.7)	47 (4.9)	231 (35.9)	299 (14.6)
**Religion, n (%)**				
Other/ Non-believer	97 (21.6)	110 (11.5)	65 (10.1)	272 (13.2)
Christian	282 (62.8)	747 (78.5)	525 (81.7)	1554 (76.0)
Muslim	70 (15.6)	95 (10.0)	53 (8.2)	220 (10.8)
**Alcohol consumption, n (%)**				
Non-drinker	66 (14.7)	323 (33.9)	326 (50.7)	715 (34.9)
Moderate drinking	79 (17.6)	197 (20.7)	130 (20.2)	406 (19.9)
Hazardous consumption	304 (67.7)	432 (45.4)	187 (29.1)	923 (45.2)
**Tobacco consumption, n (%)**				
Non-smoker	89 (19.8)	825 (86.7)	546 (84.9)	1460 (71.4)
Smoker	360 (80.2)	127 (13.3)	97 (15.1)	584 (28.6)
**HIV infection, n (%)**				
No	433 (96.4)	827 (86.9)	504 (78.4)	1764 (86.3)
Yes	16 (3.6)	125 (13.1)	139 (21.6)	280 (13.7)

IQR: interquartile range

DU: drug user; FSW: female sex worker; MSM: men who have sex with men

### Prevalence of psychological distress

The median Kessler score was 14 (IQR = [10–19]) for MSM, 18 (IQR = [13–23]) for FSW and 21 (IQR = [16–27]) for DU. About two-thirds (n = 1,244; 61.0%) of the participants did not have PD. The overall prevalence of mild PD among the three populations was 19.9% (95%CI = [18.3–21.8]) and was 19.2% (95%CI = [17.5–20.9]) for the severe/moderate PD. The prevalence of PD was statistically different in the three groups (p-value <0.001). Among DU, the prevalence of mild PD was 23.4%, 95%CI = [20–28] and 32.1%, 95%CI = [28–37] had severe/moderate PD. Severe/moderate PD was reported among one FSW in five (19.0%; 95%CI = [17–22]). The relationships between sociodemographic and clinical characteristics and PD are presented in Table 2.

**Table 2:** Associations between psychological distress^a^ and sociodemographic and clinical characteristics among key populations (N = 2044).

	Psychological distress			
	No	Mild	Severe/Moderate	P-value
**Prevalence, n (%)**	1244 (60.9)	408 (19.9)	392 (19.2)	
**Age (years), median [IQR]**	24 [21–30]	26 [21–33]	28 [22.5–34]	<0.001^*^
**Marital status, n (%)**				<0.001^**^
Not married	1081 (62.7)	334 (19.4)	308 (17.9)	
Married/Living with a partner	163 (50.8)	74 (23.0)	84 (26.2)	
**Education level, n (%)**				<0.001^**^
Primary school	292 (45.4)	164 (25.5)	187 (29.1)	
Secondary school	725 (65.8)	208 (18.9)	169 (15.3)	
College/University	227 (76.0)	36 (12.0)	36 (12.0)	
**Religion, n (%)**				0.003^**^
Other/ Non-believer	136 (50.0)	67 (24.6)	69 (25.4)	
Christian	973 (62.6)	297 (19.1)	284 (18.3)	
Muslim	135 (61.9)	44 (20.2)	39 (17.9)	
**Alcohol consumption, n (%)**				<0.001^**^^**^
Non-drinker	502 (70.2)	117 (16.4)	96 (13.4)	
Moderate drinking	244 (60.1)	83 (20.4)	79 (19.5)	
Hazardous consumption	498 (54.0)	208 (22.5)	217 (23.5)	
**Tobacco consumption, n (%)**				<0.001^**^
Non-smoker	1069 (63.3)	333 (19.7)	288 (17.0)	
Smoker	175 (49.4)	75 (21.2)	104 (29.4)	
**HIV infection, n (%)**				0.010^**^
No	1075 (60.9)	366 (20.8)	323 (18.3)	
Yes	169 (60.4)	42 (15.0)	69 (24.6)	
**Key population**				<0.001^**^
DU	200 (44.5)	105 (23.4)	144 (32.1)	
FSW	548 (57.6)	223 (23.4)	181 (19.0)	
MSM	496 (77.1)	80 (12.5)	67 (10.4)	

^a^ Psychological distress was measured with the Kessler Psychological Distress Scale (10). Total Kessler score <20: No Psychological distress, 20–24: Mild disorder, 25–50: Moderate/Severe disorder.

^*^ Kruskall-Wallis test

^**^ Chi-square test

IQR: interquartile range

DU: drug user; FSW: female sex worker; MSM: men who have sex with me

### Factors associated with psychological distress

Results of ordinal logistic regression models among key population are presented in Table 3.

**Table 3:** Factors associated with psychological distress among key population in Togo (Partial proportional odds model^#^).

(Psychological distress: 1 = Severe/Moderate, 2 = Mild, 3 = No)						
	Univariable			Multivariable		
	1 vs (2 and 3)	(1 and 2) vs 3	P-value	1 vs (2 and 3)	(1 and 2) vs 3	P-value
	OR (95% CI)	OR (95% CI)		aOR (95% CI)	aOR (95% CI)	
**Age (years)**						
< 25	Reference		**<0.001**	Reference		**0.026**
≥ 25	1.77 (1.48–2.11)	1.77 (1.48–2.11)		1.24 (1.02–1.50)	1.24 (1.02–1.50)	
**Marital status**			**<0.001**			**-**
Not married	Reference			-	-	
Married/Living with a partner	1.63 (1.29–2.04)	1.63 (1.29–2.04)		-	-	
**Education level**			**<0.001**			**<0.001**
Primary school	Reference			Reference		
Secondary	0.44 (0.36–0.52)	0.44 (0.36–0.52)		0.52 (0.42–0.64)	0.52 (0.42–0.64)	
College/University	0.27 (0.20–0.37)	0.27 (0.20–0.37)		0.46 (0.32–0.64)	0.46 (0.32–0.64)	
**Religion**			**<0.001**			**-**
Other/ Non-believer	Reference			-	-	
Christian	0.62 (0.48–0.78)	0.62 (0.48–0.78)		-	-	
Muslim	0.63 (0.44–0.88)	0.63 (0.44–0.88)		-	-	
**Alcohol consumption**			**<0.001**			**<0.001**
Non-drinker	Reference			Reference		
Moderate drinking	1.56 (1.22–2.0)	1.56 (1.22–2.0)		1.28 (0.98–1.65)	1.28 (0.98–1.65)	
Hazardous consumption	2.0 (1.64–2.45)	2.0 (1.64–2.45)		1.52 (1.22–1.87)	1.52 (1.22–1.87)	
**Tobacco consumption**			**<0.001**			**-**
Non-smoker	Reference					
Smoker	1.84 (1.46–2.32)	1.48 (1.22–1.80)		-	-	
**HIV infection**			**0.004**			**<0.001**
No	Reference			Reference		
Yes	1.46 (1.08–1.96)	1.02 (0.79–1.32)		1.80 (1.31–2.48)	1.25 (0.94–1.65)	
**Key population**			**<0.001**			**<0.001**
DU	Reference			Reference		
FSW	0.56 (0.45–0.69)	0.56 (0.45–0.69)		0.55 (0.43–0.68)	0.55 (0.43–0.68)	
MSM	0.23 (0.18–0.30)	0.23 (0.18–0.30)		0.33 (0.24–0.44)	0.33 (0.24–0.44)	

^#^ For nearly all variables, the left 2 columns are the same, so are the right 2 columns, because the assumption of proportional odds was met for those variables and therefore we used the same value for both columns. (Only the OR for tobacco smoker and HIV infection were different.)

In univariable analysis, age, marital status, education level, religion, alcohol consumption, tobacco consumption, HIV infection status and the group of key population were associated with PD (Table 3).

In multivariable analysis, after adjustment on the other variables, respondents who were 25 years old and older, were more likely to have severe/moderate or mild PD than respondents who were younger (p = 0.026). HIV positive serological status was a risk factor for PD (aOR = 1.80; 95%CI [1.31–2.48]). The odds of PD were higher among hazardous drinkers (aOR = 1.52; 95%CI = [1.22–1.87]) compared to non-drinkers. Those with secondary school level or with college/university level were less likely to have severe/moderate or mild PD compared to those who stopped in primary school (p<0.001). Compared to DU, FSW (aOR = 0.55; 95%CI = [0.43–0.68]) and MSM (aOR = 0.33; 95%CI = [0.24–0.44]) were less likely to have severe/moderate or mild PD.

## Discussion

Among MSM, FSW, and DU in Togo, the overall prevalence of mild and severe/moderate psychological distress was 19.9% and 19.2% respectively. DU had higher level of psychological distress compared with FSW and MSM (p<0.001). Also, among the three populations, participants with HIV infection were more likely to experience psychological distress than their non-infected counterparts. Factors associated with psychological distress other than HIV infection were greater age, lower education level and hazardous alcohol consumption.

We did not have national data or data among key populations in Togo to which we could compare our findings. Other studies using K-10 were conducted in Africa. In the general population in South Africa (n = 25850), 23.9% of study participants reported psychological distress (K-10≥20) [34]. Hazardous alcohol drinking (OR = 1.79) as well as HIV positive status (OR = 4.76) were associated with psychological distress as observed in our study. In another cross-sectional study among 180 patients conducted in drug rehabilitation centers in Nepal which used K-6 as psychological distress measurement tool, the prevalence of high psychological distress was 51.1% [35]. Factors associated with psychological distress were age, education level, severity of drug abuse. Among Latino and African American MSM living in the USA, 22% had moderate/severe psychological distress using the K-10 scale [36]. This prevalence is similar to the overall moderate/severe psychological distress prevalence in our study among the three key populations (19.0%). However, this estimate was higher than that reported among MSM in our study (10.4%). There is a gap between the percentages of PD in other countries and in our study mainly because of the age differences. Indeed, the population considered in our study is relatively young (median age = 25 years) unlike in other studies [35,36]. Also, different tools have been used in the other studies to measure PD as the Patient Health Questionnaire (PHQ) [19], the Center for Epidemiologic Studies Depression Scale (CES-D) [40] and K-6 [35].

Several studies in developed countries have reported that alcohol or drug consumption are closely associated with psychological distress. This is consistent with our study regarding alcohol consumption. Binge drinking or cocaine consumption were associated with psychological distress among Latino and African American in the USA [36].

Among 280 HIV infected patients in our study, 39.6% had psychological distress (K-10 scale>20). In a Nigerian study among 117 people living with HIV/AIDS recruited in a teaching hospital, 47.9% participants had psychological distress (K-10 scale>20) [37] and alcohol use was associated with PD. Similar results were reported in Ethiopia where an association between PD and alcohol use disorder was reported (OR = 1.90) [38].

A limited number of studies among key populations used the Kessler scale. However mental health was explored in these populations with tools such as the PHQ and the CES-D. A study in India among MSM (n = 1176) measured the prevalence of depression with PHQ-2 tool. Having had an STI in the six months preceding the study, being HIV positive, not having used a condom during sexual intercourse were associated with depression [19]. In the USA, a cross-sectional survey was conducted among Lesbian, Gay male, and Bisexual (LGB) older adults, aged 50 and older (n = 2439). This study investigated depression among LGB with the CES-D tool and the findings revealed that lifestyle, financial barriers, obesity were factors which accounted for poor general health, and depression among LGB older adults [39]. Another study conducted in the USA among black MSM (n = 197) used the CES-D to measure depression. This study has also reported a link between alcohol consumption and depressive symptoms [40].

Little is known about mental health among key populations in sub-Saharan Africa, and to our knowledge this study is the first one which has assessed psychological distress among these groups using an international validated tool (K-10). This tool has been validated in the African context in South Africa in general population [34]. In addition, we used data from a nationally representative study (n = 2044) including three main groups of key populations, allowing for the generalization of our findings. Finally, our study provided useful information on factors associated with PD among key populations in order to design specific interventions within these populations.

Our study has some limitations. First, we worked on three populations, but it is possible that an FSW may be a DU or an MSM may also be a DU. However, to classify the population, we took into account only the entry point. Since our study was based on self-reporting, social desirability bias cannot be ruled out. This bias could have underestimated the prevalence rates of PD. Also, as we conducted a cross-sectional study, causality between the factors identified and PD could not be addressed. Other factors such as an employment status were not included although they could potentially affect psychological distress, as reported in a study conducted among MSM in South Africa [34]. Finally, there was no medical evaluation in the present study.

## Conclusion and recommendations

This is the first study conducted among a large, nationally representative sample of key populations in Togo. The prevalence of PD is high among Key Population in Togo and was associated to HIV infection. Mental health care such as social support groups must be integrated within health and prevention programs dedicated to key populations in Togo in order to promote a holistic perspective of their health. In order to confirm the high prevalence of PD, studies in the general population or comparative studies are needed. Also, since PD a multifactorial process, it would be useful to couple a qualitative study with a quantitative study.

## Supporting information

S1 Data (XLSX)

S2 Data (XLSX)

S3 Data (XLSX)

## List of abbreviations

95% CI: 95% confidence interval
aOR: adjusted Odds Ratio
IQR: interquartile range
PD: psychological distress

## References

[1] Organisation Mondiale de la Santé. Mental health: strengthening our response [Internet]. 2018 [cited 2019 Apr 8]. Available from: https://www.who.int/health-topics/news-room/fact-sheets/detail/mental-health-strengthening-our-response

[2] Whiteford HA, Degenhardt L, Rehm J, Baxter AJ, Ferrari AJ, Erskine HE, et al. Global burden of disease attributable to mental and substance use diso—PubMed—NCBI [Internet]. [cited 2019 Apr 19]. Available from: https://www.ncbi.nlm.nih.gov/pubmed/23993280.

[3] Prince M, Patel V, Saxena S, Maj M, Maselko J, Phillips MR, et al. No health without mental health.—PubMed—NCBI [Internet]. [cited 2019 Apr 19]. Available from: https://www.ncbi.nlm.nih.gov/pubmed/17804063.

[4] Banque Mondiale. Mental health [Internet]. World Bank. 2018 [cited 2019 Apr 8]. Available from: http://www.worldbank.org/en/topic/mental-health

[5] Hassanzadeh J, Asadi-Lari M, Ghaem H, Kassani A, Niazi M, Menati R. The Association of Poor Mental Health Status and Sociocultural Factors in Men: A Population-Based Study in Tehran, Iran. Am J Mens Health. 2018 1;12(1):96–103. 10.1177/1557988316630720 26883076 PMC5734542

[6] Heather Sipsma, Angela Ofori-Atta, Maureen Canavan, Isaac Osei-Akoto, Christopher Udry, and Elizabeth H Bradley. Poor mental health in Ghana: who is at risk? [Internet]. [cited 2019 Apr 19]. Available from: https://www.ncbi.nlm.nih.gov/pmc/articles/PMC3620375 10.1186/1471-2458-13-288 PMC3620375 23547846

[7] UNAIDS. TERMINOLOGY GUIDELINES [Internet]. 2015 [cited 2019 Apr 8]. Available from: http://www.unaids.org/sites/default/files/media_asset/2015_terminology_guidelines_en.pdf

[8] Sandfort TG, de Graaf R, Bijl RV, Schnabel P. Same-sex sexual behavior and psychiatric disorders: Findings from the Netherlands Mental Health Survey and Incidence Study (NEMESIS). Archives of general psychiatry. 2001;58(1):85–91. 10.1001/archpsyc.58.1.85 11146762

[9] Luo Zin, Feng T, Fu H, Yang T. Lifetime prevalence of suicidal ideation among men who have sex with men: a meta-analysis. BMC Psychiatry [Internet]. 2017 Dec 21 [cited 2019 Apr 8];17. Available from: https://www.ncbi.nlm.nih.gov/pmc/articles/PMC5740861/10.1186/s12888-017-1575-9 PMC5740861 29268723

[10] Christine T Rael, Alissa Davis. Depression and key associated factors in female sex workers and women living with HIV/AIDS in the Dominican Republic [Internet]. 2017. Available from: https://journals.sagepub.com/doi/abs/10.1177/0956462416651374 10.1177/0956462416651374 PMC5114170 27189491

[11] Savin-Williams RC. Verbal and physical abuse as stressors in the lives of lesbian, gay male, and bisexual youths: associations with school problems, running away, substance abuse, prostitution, and suicide. Journal of consulting and clinical psychology. 1994;62(2):261 10.1037//0022-006x.62.2.261 8201062

[12] Collins PY, Holman AR, Freeman MC, Patel V. What is the relevance of mental health to HIV/AIDS care and treatment programs in developing countries? A systematic review. AIDS (London, England). 2006;20(12):1571. 10.1097/01.aids.0000238402.70379.d4 PMC2801555 16868437

[13] Hutton HE, Lyketsos CG, Zenilman JM, Thompson RE, Erbelding EJ. Depression and HIV risk behaviors among patients in a sexually transmitted disease clinic. American Journal of Psychiatry. 2004;161(5):912–914. 10.1176/appi.ajp.161.5.912 15121659

[14] Safren SA, Thomas BE, Mimiaga MJ, Chandrasekaran V, Menon S, Swaminathan S, et al Depressive symptoms and human immunodeficiency virus risk behavior among men who have sex with men in Chennai, India. Psychology, health & medicine. 2009;14(6):705–715. 10.1080/13548500903334754 PMC3640638 20183543

[15] Klein H. Depression and HIV risk taking among men who have sex with other men and who use the internet to find Partners for Unprotected sex. Journal of gay & lesbian mental health. 2014;18(2):164–189. 26877831 10.1080/19359705.2013.834858 PMC4752171

[16] Prajapati AC, Parikh S, Bala DV. A study of mental health status of men who have sex with men in Ahmedabad city. Indian journal of psychiatry. 2014;56(2):161 10.4103/0019-5545.130498 24891704 PMC4040064

[17] van Griensven F, van Wijngaarden JW de L. A review of the epidemiology of HIV infection and prevention responses among MSM in Asia. Aids. 2010;24:S30–S40. 10.1097/01.aids.0000390087.22565.b4 20926926

[18] No health without mental health.—PubMed—NCBI [Internet]. [cited 2019 Apr 8]. Available from: https://www.ncbi.nlm.nih.gov/pubmed/17804063

[19] Patel SK, Prabhakar P, Saggurti N. Factors Associated with Mental Depression among Men Who Have Sex with Men in Southern India. Health. 2015;7(09):1114.

[20] Teclessou JN, Akakpo S, Gbetoglo D, Koumagnanou G, Singo A, Pitché P. HIV prevalence and behavioral studies among female sex workers in Togo in 2015. Bull Soc Pathol Exot. 2017 Oct;110(4):270–5. 10.1007/s13149-017-0565-y 28660604

[21] Teclessou JN, Akakpo SA, Ekouevi KD, Koumagnanou G, Singo-Tokofai A, Pitche PV. Evolution of HIV prevalence and behavioral factors among MSM in Togo between 2011 and 2015. Pan Afr Med J. 2017;28:191 10.11604/pamj.2017.28.191.11285 29599889 PMC5873283

[22] Kessler RC, Andrews G, Colpe LJ, Hiripi E, Mroczek DK, Normand S-L, et al Short screening scales to monitor population prevalences and trends in non-specific psychological distress. Psychological medicine. 2002;32(6):959–976. 10.1017/s0033291702006074 12214795

[23] Myer L, Stein DJ, Grimsrud A, Seedat S, Williams DR. Social determinants of psychological distress in a nationally-representative sample of South African adults. Social science & medicine. 2008;66(8):1828–1840. 18299167 10.1016/j.socscimed.2008.01.025 PMC3203636

[24] Peltzer K, Pengpid S, Skaal L. Prevalence of psychological distress and associated factors in urban hospital outpatients in South Africa. South African Journal of Psychiatry. 2012;18(1):10–15.

[25] Babor TF, de la Fuente JR, Saunders J, Grant M. The Alcohol Use Disorders Identification Test: Guidelines for use in. 2001;

[26] WHO | Surveillance and monitoring [Internet]. WHO [cited 2019 Mar 5]. Available from: http://www.who.int/tobacco/surveillance/en/

[27] Amon J, Brown T, Hogle J, MacNeil J, Magnani R, Mills S, et al. Behavioral Surveillance Surveys BSS. Guidelines for repeated behavioral surveys in populations at risk of HIV. 2000;

[28] Andrews G, Slade T. Interpreting scores on the Kessler psychological distress scale (K10). Australian and New Zealand journal of public health. 2001;25(6):494–497. 10.1111/j.1467-842x.2001.tb00310.x 11824981

[29] Fiellin DA, Reid MC, O’connor PG. Screening for alcohol problems in primary care: a systematic review. Archives of internal medicine. 2000;160(13):1977–1989. 10.1001/archinte.160.13.1977 10888972

[30] Foxcroft DR, Smith LA, Thomas H, Howcutt S. Accuracy of alcohol use disorders identification test for detecting problem drinking in 18–35 year-olds in England: method comparison study. Alcohol and alcoholism. 2014;50(2):244–250. 10.1093/alcalc/agu095 25534931

[31] Gache P, Michaud P, Landry U, Accietto C, Arfaoui S, Wenger O, et al The Alcohol Use Disorders Identification Test (AUDIT) as a screening tool for excessive drinking in primary care: reliability and validity of a French version. Alcoholism: Clinical and Experimental Research. 2005;29(11):2001–2007. 10.1097/01.alc.0000187034.58955.64 16340457

[32] Peterson B, Harrell FE Jr. Partial proportional odds models for ordinal response variables. Journal of the Royal Statistical Society: Series C (Applied Statistics). 1990;39(2):205–217.

[33] Fullerton AS, Xu J. The proportional odds with partial proportionality constraints model for ordinal response variables. Social science research. 2012;41(1):182–198. 10.1016/j.ssresearch.2011.09.003 23017706

[34] Mthembu JC, Mabaso MLH, Khan G, Simbayi LC. Prevalence of psychological distress and its association with socio-demographic and HIV-risk factors in South Africa: Findings of the 2012 HIV prevalence, incidence and behaviour survey. SSM-population health. 2017;3:658–662. 10.1016/j.ssmph.2017.07.009 29349254 PMC5769074

[35] Gyawali B, Choulagai BP, Paneru DP, Ahmad M, Leppin A, Kallestrup P. Prevalence and correlates of psychological distress symptoms among patients with substance use disorders in drug rehabilitation centers in urban Nepal: a cross-sectional study. BMC psychiatry. 2016;16(1):314 10.1186/s12888-016-1003-6 27609330 PMC5015326

[36] Zaller N, Yang C, Operario D, Latkin C, McKirnan D, O’Donnell L, et al Alcohol and cocaine use among Latino and African American MSM in 6 US cities. Journal of substance abuse treatment. 2017;80:26 10.1016/j.jsat.2017.06.003 28755769 PMC5839147

[37] Igwe MN, Ndukuba AC, Olose EO, Obayi NO, Ajayi NA, Odo A, et al Psychological distress and quality of life of people living with HIV/AIDS in a Nigerian teaching hospital. Mental Health, Religion & Culture. 2016;19(9):961–971.

[38] Soboka M, Tesfaye M, Feyissa GT, Hanlon C. Alcohol use disorders and associated factors among people living with HIV who are attending services in south west Ethiopia. BMC research notes. 2014;7(1):828. 25417542 10.1186/1756-0500-7-828 PMC4289332

[39] Fredriksen-Goldsen KI, Emlet CA, Kim H-J, Muraco A, Erosheva EA, Goldsen J, et al The Physical and Mental Health of Lesbian, Gay Male, and Bisexual (LGB) Older Adults: The Role of Key Health Indicators and Risk and Protective Factors. Gerontologist. 2013 8 1;53(4):664–75. 10.1093/geront/gns123 23034470 PMC3709843

[40] Reisner SL, Mimiaga MJ, Skeer M, Bright D, Cranston K, Isenberg D, et al Clinically significant depressive symptoms as a risk factor for HIV infection among black MSM in Massachusetts. AIDS and Behavior. 2009;13(4):798–810. 10.1007/s10461-009-9571-9 19462228 PMC3947411

