# Supplementary material for: Prevalence and factors associated with psychological distress among key populations in Togo, 2017
Source: PLoS One. 2020 Apr 16;15(4):e0231726. doi: 10.1371/journal.pone.0231726 (PMC7162496; doi:10.1371/journal.pone.0231726)
Supplement: Attachment — Submitted filename: Review_Factors associated with PD among KPs in Togo.docx [file pone.0231726.s004.docx]

**Review for article titled**: Prevalence and Factors Associated with Psychological Distress among Key Populations in Togo, 2017

Overall, the paper reads well, congratulations!

I have minor comments that I think need to be addressed:

**Abstract**

1. The write up has several grammatical errors that should be worked on.
2. Use of abbreviations should come after you have spelled the long form earlier. In the abstract, the author has used the abbreviation PD for the first time before it is written in full.
3. In reporting factors associated with PD, the author could choose to report such factors in groups; that is those that increase the risk and those that reduce the risk.
4. The conclusion sounds more like a recommendation. I advise that you give concluding statements based on your findings. You may want to title the section as Conclusion and Recommendation and include a sentence on recommendations.

**Introduction:**

1. When making reference of previous studies, there is no need to report estimates and their 95% confidence intervals.
2. A statement, .... “*poor mental status has been identified as a key contributor to HIV epidemic among key populations*,” requires a citation.

**Results:**

1. The statement, “*the prevalence of psychological distress was statistically different in the three groups*” lacks supporting statistical evidence to substantiate it.
2. Table 2:
   a. All percentages should be row percentages to make sense of the relationships between variables
   b. The p-value reported for the relationship between age and psychological distress need to be described whether it is a p-for trend or not. If it is not a p-value for trend, then there is also a need to mentions which comparison is being made.

**Discussion:**

1. When referencing previous studies, there is no need to report the estimates and their 95% confidence intervals.
2. Grammatically, use “a” and “an” as appropriately. FSW should be preceded by “an” and not “a”…similarly, it is “an MSM” and not “a MSM”
3. In the limitations of your study, you have pointed out some potential bias that could have been introduced in your study. However, you did not show or describe the potential impact of the introduced bias to your results.
4. One of the limitations you have mentioned is the cross-sectional nature of your study pointing that, you cannot elicit causality from this design. Does this mean the findings are of no use? Can you mention how can we make use of your findings despite the lack of causality?
5. The last paragraph of your discussion presents recommendations. I advise it be moved to an appropriate section.

**Conclusion:**

1. Your conclusion presents recommendations. I would advise you change the title of the section to Conclusion and Recommendation and then include concluding remarks as well as recommendations.
