# Supplementary material for: Prevalence and factors associated with psychological distress among key populations in Togo, 2017
Source: PLoS One. 2020 Apr 16;15(4):e0231726. doi: 10.1371/journal.pone.0231726 (PMC7162496; doi:10.1371/journal.pone.0231726)
Supplement: Attachment — Submitted filename: Response_to_Reviewers.docx [file pone.0231726.s005.docx]

**Response to reviewers**

**Reviewer #1:** Thanks for the opportunity to read and learn about your interesting work. May I offer the following suggestions.

**Authors: We thank the reviewer for this encouraging comment.**

1. *It is important to be clear about the direction of associations in key places like the Abstract and in the first paragraph of Discussion. I think most of what you say in the Results section of the Abstract is okay *except* regarding education. I recommend you end the list of factors with "hazardous alcohol consumption (aOR and 95% CI are listed here) were associated with PD." Then start a new sentence saying something like "People with secondary (aOR and 95% CI here) or higher (aOR and CI) education levels were LESS likely to report PD."*

*Then in the first paragraph of Discussion section (page 11), you should specify GREATER age and LOWER education level.*

**Authors: We thank the reviewer for these comments which have been taken into consideration in the abstract and discussion sections.**

HIV prevalence was 13.7% (95%CI = [12.2-15.2]). High age (25 years and over) [aOR = 1.24 (95% CI: 1.02–1.50)], being HIV positive [aOR = 1.80 (95% CI: 1.31–2.48)] and hazardous alcohol consumption [aOR = 1.52 (95% CI: 1.22–1.87)] were risks factors associated with PD. People with secondary [aOR = 0.52 (95% CI: 0.42-0.64)] or higher [aOR = 0.46 (95% CI: 0.32-0.64)] education levels were protective factors associated with PD.

**Discussion (page 10)**

Factors associated with psychological distress other than HIV infection were greater age, lower education level and hazardous alcohol consumption.

2. *Where you refer to bivariate and multivariate methods, my understanding is these should be labeled Univariable and Multivariable (ending with -able instead of -ate). See https://www.ncbi.nlm.nih.gov/pmc/articles/PMC3679183/ for discussion.*

**Authors: We thank the reviewer for these comments. All the words have been relabeled (bivariate relabeled univariable and multivariate relabeled multivariable).**

Descriptive statistics, univariable and multivariable ordinal regression models were used for analysis.

For model building, characteristics that had a p-value <0.20 in univariable analysis were considered for the full multivariable models, which were subsequently finalized using a stepwise, backward elimination approach (p-value <0.05).

In univariable analysis, age, marital status, education level, religion, alcohol consumption, tobacco consumption, HIV infection status and the group of key population were associated with PD (Table 3).

In multivariable analysis, after adjustment on the other variables, respondents who were 25 years old and older, were more likely to have severe/moderate or mild PD than respondents who were younger (p=0.026).

3. *There are several places where you splice two sentences together with the word "and." Probably better to separate to 2 sentences, or use a semicolon instead of the "and." e.g., near bottom of page 2 after citations (11-13).*

**Authors: We thank the reviewer for these comments which have been taken into consideration in the document.**

4. *Specific:
   Page 1 Abstract, last line in Methods section, should "model" have an -s on it ? Also, not sure if you can use abbreviation PD without introducing it, even though it appears later in the sentence in the name of the scale.*

**Authors: We thank the reviewer for these comments which have been taken into consideration in the document.**

## Methods

Psychological distress (PD) was assessed with the Kessler Psychological Distress Scale. A blood sample was taken to test for HIV. Descriptive statistics, univariable and multivariable ordinal regression models were used for analysis.

5. *Regarding Results section of Abstract, see note below about so many zeros after decimal points. for median and IQR, delete the ".0" because you are using only integer values. For the percentages, some of the ".0" are actually not correct. 19.0% should be 19.2% (392/2044). Although I did not calculate the CI's, those too are not correct and you will have to calculate those yourself (where it says 18.0-22.0 and 17.0-21.0).*

**Authors: We thank the reviewer for these comments which have been taken into consideration in the document.**

## Results

The overall prevalence of mild PD among the three populations was 19.9% (95%CI= [18.3-21.8]) and was 19.2% (95%CI= [17.5-20.9]) for severe/moderate PD. HIV prevalence was 13.7% (95%CI = [12.2-15.2]).

6. *page 2, 2nd paragraph. I would change the 1st 2 sentences around a bit: "Some population subgroups, such as those at higher risk of HIV, are particularly burdened with mental health issues. According to UNAIDS (etc …)"*

**Authors: We thank the reviewer for these comments which have been taken into consideration in the document.**

Some population subgroups, such as those at higher risk of HIV, are particularly burdened with mental health issues. According to the UNAIDS, gay men and other men who have sex with men (MSM), female sex workers (FSW) and their clients, transgender people, people who inject drugs and prisoners and other incarcerated people are the main key population groups (7).

7. *page 2, 8th line from bottom, insert "the" so it says "key contributor to the HIV epidemic"
   page 3 line 7, add "of" so it says "prevalence of and factors associated with"*

**Authors: We thank the reviewer for these comments which have been taken into consideration in the document.**

Moreover, poor mental health has been identified as a key contributor to the HIV epidemic among key populations.

The aim of this study was to assess the prevalence of and factors associated with PD among MSM, FSW and DU in Togo in 2017.

8. *Need just a little more info on what "passives, actives" refers to - is it sexual position?*

**Authors: We thank the reviewer for these comments. These two notions refer to sexual positions: the active designates the one who penetrates and the passive, the penetrated.**

9. *Last paragraph on Kessler PD Scale - some of this text is repeated on the next page; delete one or the other.*

**Authors: We thank the reviewer for these comments which have been taken into consideration in the document.**

10. *Page 4 -- should be only one L in syphilis. Then 2 lines down, delete s on "tests"
    next paragraph is where Kessler PD repeat occurs. Also in that paragraph, change "allows to categorize" to "allows categorization of."*

**Authors: We thank the reviewer for these comments which have been taken into consideration in the document.**

Two HIV rapid tests were performed using SD Biolane Duo VIH/Syphilis rapid test kits. For each participant, a 4 mL blood sample was collected and a third test, ImmunoComb II VIH1-2 Comfirm (Orgenics Ltd, Israël) was used for confirmation. All biological test analyses were completed in the principal HIV laboratory research unit, the Molecular Biology Laboratory (CBMI) at the University of Lomé.

## Scores and operational definitions

***Psychological Distress***

The score obtained from the scale allows categorization of participants into four categories of PD: severe (score ≥ 30), moderate (score: 25-29), mild (score: 20-24) and none (score < 20) (28).

11. *page 5: along with "latent" mention that PO model is used for *ordinal* outcome variables.*

**Authors: We thank the reviewer for these comments which have been taken into consideration in the document.**

We considered Partial Proportional Odds model for the dependent variable Psychological Distress (PD).

- The Proportional Odds (PO) model is appropriate when the response categories are based on one or more latent response variable.

12. *With backward elimination approach, what was the criterion for elimination? e.g., P<0.05, 0.01?*

**Authors: We thank the reviewer for these comments. The criterion for elimination was p<0.05 for the multivariable model.**

For model building, characteristics that had a p-value <0.20 in univariable analysis were considered for the full multivariable models, which were subsequently finalized using a stepwise, backward elimination approach (p-value <0.05).

13. *page 6: first sentence regarding Kessler scores, leave off all the zeros after decimal for median and IQR scores, since these Kessler score values are always integers. On the other hand, in the next sentence some of the zeros after decimal point in the percentages are not correct. 20.0% is correct, but 61.0% should be 60.9%, and 19.0% should be 19.2%. You will need to recalculate your 95% CI's in this sentence and the rest of this paragraph - these should not all be ending with ".0%".*

**Authors: We thank the reviewer for these comments which have been taken into consideration in the document.**

## Prevalence of psychological distress

The median Kessler score was 14 (IQR = [10-19]) for MSM, 18 (IQR = [13-23]) for FSW and 21 (IQR= [16-27]) for DU. About two-thirds (n=1,244; 61.0%) of the participants did not have psychological distress. The overall prevalence of mild psychological distress among the three populations was 19.9% (95%CI= [18.3-21.8]) and was 19.2% (95%CI= [17.5-20.9]) for the severe/moderate psychological distress. The prevalence of psychological distress was statistically different in the three groups (p-value <0.001). Among DU, the prevalence of mild psychological distress was 23.4%, 95%CI = [20-28] and 32.1%, 95%CI = [28-37] had severe/moderate psychological distress. Severe/moderate psychological distress was reported among one FSW in five (19.0%; 95%CI = [17-22]). The relationships between sociodemographic and clinical characteristics and psychological distress are presented in Table 2.

14. *I don't think you need Figure 1. Instead, just add the key population groups as another variable at the bottom of Table 2.*

*In Table 2, you need to make the same corrections to percentages (first row only) as in the text, namely change 61.0 to 60.9 and 19.0 to 19.2. On age, delete the ".0"s since all these numbers are integers only. Throughout the rest of Table 2, I think the ROW totals should add to 100% rather than the column totals. That helps the reader quickly approximate the odds ratios you will be presenting in Table 3.*

**Authors: We thank the reviewer for these comments which have been taken into consideration in the document.**

**Table 2: Associations between psychological distress^a^ and sociodemographic and clinical characteristics among key populations (N=2044)**

|  | Psychological distress | | |  |
| --- | --- | --- | --- | --- |
|  | No | Mild | Severe/Moderate | P-value |
| **Prevalence, n (%)** | 1244 (60.9) | 408 (19.9) | 392 (19.2) |  |
| **Age (years), median [IQR]** | 24 [21-30] | 26 [21-33] | 28 [22.5-34] | **<0.001^*^** |
| **Marital status, n (%)** |  |  |  | **<0.001^**^** |
| Not married | 1081 (62.7) | 334 (19.4) | 308 (17.9) |  |
| Married/Living with a partner | 163 (50.8) | 74 (23.0) | 84 (26.2) |  |
| **Education level, n (%)** |  |  |  | **<0.001^**^** |
| Primary school | 292 (45.4) | 164 (25.5) | 187 (29.1) |  |
| Secondary school | 725 (65.8) | 208 (18.9) | 169 (15.3) |  |
| College/University | 227 (76.0) | 36 (12.0) | 36 (12.0) |  |
| **Religion, n (%)** |  |  |  | **0.003^**^** |
| Other/ Non-believer | 136 (50.0) | 67 (24.6) | 69 (25.4) |  |
| Christian | 973 (62.6) | 297 (19.1) | 284 (18.3) |  |
| Muslim | 135 (61.9) | 44 (20.2) | 39 (17.9) |  |
| **Alcohol consumption, n (%)** |  |  |  | **<0.001^**^** |
| Non-drinker | 502 (70.2) | 117 (16.4) | 96 (13.4) |  |
| Moderate drinking | 244 (60.1) | 83 (20.4) | 79 (19.5) |  |
| Hazardous consumption | 498 (54.0) | 208 (22.5) | 217 (23.5) |  |
| **Tobacco consumption, n (%)** |  |  |  | **<0.001^**^** |
| Non-smoker | 1069 (63.3) | 333 (19.7) | 288 (17.0) |  |
| Smoker | 175 (49.4) | 75 (21.2) | 104 (29.4) |  |
| **HIV infection, n (%)** |  |  |  | **0.010^**^** |
| No | 1075 (86.4) | 366 (89.7) | 323 (82.4) |  |
| Yes | 169 (13.6) | 42 (10.3) | 69 (17.6) |  |
| **Key population** |  |  |  | **<0.001^**^** |
| DU | 200 (44.5) | 105 (23.4) | 144 (32.1) |  |
| FSW | 548 (57.6) | 223 (23.4) | 181 (19.0) |  |
| MSM | 496 (77.1) | 80 (12.5) | 67 (10.4) |  |

^a^ Psychological distress was measured with the Kessler Psychological Distress Scale (10). Total Kessler score <20: No Psychological distress, 20-24: Mild disorder, 25-50: Moderate/Severe disorder.

**^*^** Kruskall-Wallis test; **^**^** Chi-square test;

IQR: interquartile range

DU: drug user; FSW: female sex worker; MSM: men who have sex with me

15. *Page 9: Change 1st sentence to: "RESULTS OF ordinal logistic regression models (etc...)" (add s to "models")*

*Line 2, here I would say univariable analysis instead of univariate. (and note you said "bivariate" in the abstract, which I think should also say "univariable")*

*Line 3, delete the word "factors" so it just says "were associated with …". Next paragraph, change multivariate to multivariable. Add an s so it says odds in "The odd of psychological" and then change "was" to "were"*

**Authors: We thank the reviewer for these comments which have been taken into consideration in the document.**

## Factors associated with Psychological Distress

Results of ordinal logistic regression models among key population are presented in table 3.

In univariable analysis, age, marital status, education level, religion, alcohol consumption, tobacco consumption, HIV infection status and the group of key population were associated with psychological distress (Table 3).

In multivariable analysis, after adjustment on the other variables, respondents who were 25 years old and older, were more likely to have severe/moderate or mild psychological distress than respondents who were younger (p=0.026). Those with secondary school level or with college/university level were less likely to have severe/moderate or mild psychological distress compared to those who stopped in primary school (p<0.001). The odds of psychological distress were higher among hazardous drinkers (aOR=1.52; 95%CI= [1.22-1.87]) compared to non-drinkers. HIV serological status was a factor associated with psychological distress (aOR= 1.80; 95%CI [1.31-2.48]). Compared to DU, FSW (aOR= 0.55; 95%CI = [0.43-0.68]) and MSM (aOR= 0.33; 95%CI = [0.24-0.44]) were less likely to have severe/moderate or mild psychological distress.

16. *Table 3: change column headers to Univariable and Multivariable. I think you should explain in a footnote that for nearly all variables, the left 2 columns are the same, and the right 2 columns are the same, because the assumption of proportional odds was met for those variables and therefore you used the same value for both columns. (only the tobacco smoker and HIV infection OR's are different.)*

**Authors: We thank the reviewer for these comments which have been taken into consideration in the document.**

**Table 3: Factors associated with psychological distress among key population in Togo (Partial proportional Odds model) ^#^.**

|  | (Psychological distress: 1 = Severe/Moderate, 2 = Mild, 3 = No) | | | | | | |
| --- | --- | --- | --- | --- | --- | --- | --- |
|  | Univariable | | |  | Multivariable | | |
|  | 1 vs (2 and 3) | (1 and 2) vs 3 | P-value |  | 1 vs (2 and 3) | (1 and 2) vs 3 | P-value |
|  | OR (95 % CI) | OR (95 % CI) |  |  | aOR (95 % CI) | aOR (95 % CI) |  |
| **Age (years)** |  |  |  |  | |  |  |
| < 25 | Reference | | **<0.001** |  | Reference | | **0.026** |
| ≥ 25 | 1.77 (1.48-2.11) | 1.77 (1.48-2.11) |  |  | 1.24 (1.02-1.50) | 1.24 (1.02-1.50) |  |
| **Marital status** |  |  | **<0.001** |  |  |  | **-** |
| Not married | Reference | |  |  | - | - |  |
| Married/Living with a partner | 1.63 (1.29-2.04) | 1.63 (1.29-2.04) |  |  | - | - |  |
| **Education level** |  |  | **<0.001** |  |  |  | **<0.001** |
| Primary school | Reference | |  |  | Reference | |  |
| Secondary | 0.44 (0.36-0.52) | 0.44 (0.36-0.52) |  |  | 0.52 (0.42-0.64) | 0.52 (0.42-0.64) |  |
| College/University | 0.27 (0.20-0.37) | 0.27 (0.20-0.37) |  |  | 0.46 (0.32-0.64) | 0.46 (0.32-0.64) |  |
| **Religion** |  |  | **<0.001** |  |  |  | **-** |
| Other/ Non-believer | Reference | |  |  | - | - |  |
| Christian | 0.62 (0.48-0.78) | 0.62 (0.48-0.78) |  |  | - | - |  |
| Muslim | 0.63 (0.44-0.88) | 0.63 (0.44-0.88) |  |  | - | - |  |
| **Alcohol consumption** |  |  | **<0.001** |  |  |  | **<0.001** |
| Non-drinker | Reference | |  |  | Reference | |  |
| Moderate drinking | 1.56 (1.22-2.0) | 1.56 (1.22-2.0) |  |  | 1.28 (0.98-1.65) | 1.28 (0.98-1.65) |  |
| Hazardous consumption | 2.0 (1.64-2.45) | 2.0 (1.64-2.45) |  |  | 1.52 (1.22-1.87) | 1.52 (1.22-1.87) |  |
| **Tobacco consumption** |  |  | **<0.001** |  |  |  | **-** |
| Non-smoker | Reference | |  |  |  |  |  |
| Smoker | 1.84 (1.46-2.32) | 1.48 (1.22-1.80) |  |  | - | - |  |
| **HIV infection** |  |  | **0.004** |  |  |  | **<0.001** |
| No | Reference | |  |  | Reference | |  |
| Yes | 1.46 (1.08-1.96) | 1.02 (0.79-1.32) |  |  | 1.80 (1.31-2.48) | 1.25 (0.94-1.65) |  |
| **Key population** |  |  | **<0.001** |  |  |  | **<0.001** |
| DU | Reference | |  |  | Reference | |  |
| FSW | 0.56 (0.45-0.69) | 0.56 (0.45-0.69) |  |  | 0.55 (0.43-0.68) | 0.55 (0.43-0.68) |  |
| MSM | 0.23 (0.18-0.30) | 0.23 (0.18-0.30) |  |  | 0.33 (0.24-0.44) | 0.33 (0.24-0.44) |  |

aOR: adjusted Odds ratio; OR: Odds ratio

^#^ For nearly all variables, the left 2 columns are the same, so are the right 2 columns, because the assumption of proportional odds was met for those variables and therefore we used the same value for both columns. (only the tobacco smoker and HIV infection OR's are different)

17. *page 11, it is confusing to use "respectively" when there are 2 different lists. I suggest "Among MSM, FSW, and DU in Togo, the overall prevalence of mild and severe/moderate psychological distress was 20.0% and 19.2% respectively."*

**Authors: We thank the reviewer for these comments which have been taken into consideration in the document.**

# **Discussion**

Among MSM, FSW, and DU in Togo, the overall prevalence of mild and severe/moderate psychological distress was 19.9% and 19.2% respectively.

18. *2nd paragraph, could you say more about whether and why these percentages reporting PD in other countries are higher or lower than in your study? In the sentence where you cite the 23.9% in South Africa, you could say "compared to 39.2% in our study."*

**Authors: We thank the reviewer for these comments. There is a gap between the percentages of PD in other countries and in our study mainly because of the age differences. Indeed, the population considered in our study is relatively young (median age = 25 years) unlike in other studies.**

Zaller N, Yang C, Operario D, Latkin C, McKirnan D, O’Donnell L, et al. Alcohol and cocaine use among Latino and African American MSM in 6 US cities. Journal of substance abuse treatment. 2017;80:26.

Gyawali B, Choulagai BP, Paneru DP, Ahmad M, Leppin A, Kallestrup P. Prevalence and correlates of psychological distress symptoms among patients with substance use disorders in drug rehabilitation centers in urban Nepal: a cross-sectional study. BMC psychiatry. 2016;16(1):314.

*Best wishes with the manuscript and your research.*

**Authors: We thank the reviewer for this encouraging comment and for all the inputs to enhance the quality of the paper.**

**Reviewer #2:** Review for article titled: Prevalence and Factors Associated with Psychological Distress among Key Populations in Togo, 2017. Overall, the paper reads well, congratulations!
I have minor comments that I think need to be addressed:

**Authors: We thank the reviewer for this encouraging comment.**

*Abstract
1. The write up has several grammatical errors that should be worked on.*

**Authors: We thank the reviewer for these comments which have been taken into consideration in the document.**

*2. Use of abbreviations should come after you have spelled the long form earlier. In the abstract, the author has used the abbreviation PD for the first time before it is written in full.*

**Authors: We thank the reviewer for these comments which have been taken into consideration in the document.**

# **Abstract**

## Objectives

Mental health is a largely neglected issue among in Sub-Saharan Africa, especially among key populations. The aim of this study was to estimate the prevalence of psychological distress (PD) and to assess the factors associated among males who have sex with males (MSM), female sex workers (FSW) and drug users (DU) in Togo in 2017.

*3. In reporting factors associated with PD, the author could choose to report such factors in groups; that is those that increase the risk and those that reduce the risk.*

**Authors: We thank the reviewer for these comments which have been taken into consideration in the document.**

# **Abstract**

# **Results**

High age (25 years and over) [aOR = 1.24 (95% CI: 1.02–1.50)], being HIV positive [aOR = 1.80 (95% CI: 1.31–2.48)] and hazardous alcohol consumption [aOR = 1.52 (95% CI: 1.22–1.87)] were risks factors associated with PD. People with secondary [aOR = 0.52 (95% CI: 0.42-0.64)] or higher [aOR = 0.46 (95% CI: 0.32-0.64)] education levels were protective factors associated with PD.

# **Results**

# **Factors associated with Psychological Distress**

In multivariable analysis, after adjustment on the other variables, respondents who were 25 years old and older, were more likely to have severe/moderate or mild PD than respondents who were younger (p=0.026). HIV serological status was a risk factor associated with PD (aOR= 1.80; 95%CI [1.31-2.48]). The odds of PD were higher among hazardous drinkers (aOR=1.52; 95%CI= [1.22-1.87]) compared to non-drinkers. Those with secondary school level or with college/university level were less likely to have severe/moderate or mild PD compared to those who stopped in primary school (p<0.001).

*4. The conclusion sounds more like a recommendation. I advise that you give concluding statements based on your findings. You may want to title the section as Conclusion and Recommendation and include a sentence on recommendations.*

**Authors: We thank the reviewer for these comments which have been taken into consideration in the document.**

## Conclusion and Recommendations

This is the first study conducted among a large, nationally representative sample of Key Population in Togo. The prevalence of PD is high among Key Population in Togo and was associated to HIV infection. The present study indicates that mental health care must be integrated within health programs in Togo with a special focus to key populations through interventions such as social support groups.

*Introduction:
1. When making reference of previous studies, there is no need to report estimates and their 95% confidence intervals.*

**Authors: We thank the reviewer for these comments which have been taken into consideration in the document.**

*2. A statement, .... “poor mental status has been identified as a key contributor to HIV epidemic among key populations,” requires a citation.*

**Authors: We thank the reviewer for the comment. The statement above and the sentence which follows it in the text, have the same citation (11–13).**

Moreover, poor mental health has been identified as a key contributor to the HIV epidemic among key populations. Indeed, MSM who are mentally depressed engage in unprotected sex and substance abuse (11–13). Several studies have demonstrated a significant association between depressive symptoms and HIV and other sexually transmitted infections (STI) among MSM (14–16).

*Results:
1. The statement, “the prevalence of psychological distress was statistically different in the three groups” lacks supporting statistical evidence to substantiate it.*

**Authors: We thank the reviewer for the comment. We have put the p-value of the chi-square test below figure 1 but have put it now in the text since we removed the figure 1.**

The prevalence of PD was statistically different in the three groups (p-value <0.001).

*2. Table 2:*

*a. All percentages should be row percentages to make sense of the relationships between variables
b. The p-value reported for the relationship between age and psychological distress need to be described whether it is a p-for trend or not. If it is not a p-value for trend, then there is also a need to mentions which comparison is being made.*

**Authors: We thank the reviewer for these comments. All percentages have been changed into row percentages in Table 2. The p-value reported for the relationship between age and psychological distress is for a Kruskall-Wallis test.**

*Discussion:
1. When referencing previous studies, there is no need to report the estimates and their 95% confidence intervals.*

**Authors: We thank the reviewer for these comments which have been taken into consideration in the revised document.**

*2. Grammatically, use “a” and “an” as appropriately. FSW should be preceded by “an” and not “a” …similarly, it is “an MSM” and not “a MSM”*

**Authors: We thank the reviewer for these comments which have been taken into consideration in the document.**

Our study has some limitations. First, we worked on three populations, but it is possible that an FSW may be an DU or an MSM may also be an DU.

*3. In the limitations of your study, you have pointed out some potential bias that could have been introduced in your study. However, you did not show or describe the potential impact of the introduced bias to your results.*

**Authors: We thank the reviewer for these comments which have been taken into consideration in the document.**

Since our study was based on self-reporting, memory bias and social desirability bias cannot be ruled out. These bias might have had an effect on the prevalence rates of PD. Also, as we conducted a cross-sectional study, causality between the factors identified and psychological distress could not be addressed.

*4. One of the limitations you have mentioned is the cross-sectional nature of your study pointing that, you cannot elicit causality from this design. Does this mean the findings are of no use? Can you mention how can we make use of your findings despite the lack of causality?*

**Authors: We thank the reviewer for these comments. A cross sectional studies cannot prove causality.**

Like any cross-sectional study, our study cannot conclude a causality between the factors identified and psychological distress. However our results are useful to the extent that, acting on the associated factors will reduce the risk of the event occurring.

*5. The last paragraph of your discussion presents recommendations. I advise it be moved to an appropriate section.*

**Authors: We thank the reviewer for these comments which have been taken into consideration in the document. The paragraph has been moved to the conclusion.**

*Conclusion:
1. Your conclusion presents recommendations. I would advise you change the title of the section to Conclusion and Recommendation and then include concluding remarks as well as recommendations.*

**Authors: We thank the reviewer for these comments. In the revised version of the manuscript, we changed the title of the section to Conclusion and recommendations.**

# **Conclusion and Recommendations**

This is the first study conducted among a large, nationally representative sample of key populations in Togo. The prevalence of PD is high among Key Population in Togo and was associated to HIV infection. Mental health care such as social support groups must be integrated within health and prevention programs dedicated to key populations in Togo in order to promote a holistic perspective of their health. In order to confirm the high prevalence of PD, studies in the general population or comparative studies are needed. Also, since PD a multifactorial process, it would be useful to couple a qualitative study with a quantitative study.
