# Supplementary material for: Prevalence and factors associated with psychological distress among key populations in Togo, 2017
Source: PLoS One. 2020 Apr 16;15(4):e0231726. doi: 10.1371/journal.pone.0231726 (PMC7162496; doi:10.1371/journal.pone.0231726)
Supplement: Attachment — Submitted filename: Reviewers Additional Comments.docx [file pone.0231726.s006.docx]

**Prevalence and factors associated with psychological distress among key populations in Togo, 2017**

Re-review of the article:

General comment: Credit to authors for addressing the comments and making efforts to reflect the changes in the main document.

Few additional comments that you may want to consider before final production of the article:

1. Abstract:
   a. In the sub-section results line 56/57, the statement, “….were risks factors associated with PD.”, should be changed to “…**were risk factors for PD**” or “….**were factors associated with PD**.”
2. Main document:
   a. Results section:
      i. Line 231: the statement, “HIV serological status was a risk factor associated with PD…” should read, “**HIV positive serological status was a risk factor for PD**…” Having it as just HIV serological status is ambiguous.
   b. Discussion section:
      i. Line 302: “…….FSW may be an DU or an MSM may also be an DU.” Should change to “…..FSW may be **a DU** or an MSM may also be **a DU**”
      ii. Line 304/305: “These bias might have had an effect on the prevalence rates of PD.” This statement first needs to be qualified; will the biases overestimate or underestimate the prevalence? To answer this question, you will need to dissect each bias separately. Consider revision appropriately.
